# Clarifying the face of cannabis lung

**DOI:** 10.36416/1806-3756/e20240141

**Published:** 2024-09-16

**Authors:** Marialuisa Bocchino, Giacomo Sica, Roberta Lieto, Luigi Massari, Bianca Baino, Ferdinando Damato, Gaetano Rea

**Affiliations:** 1. Department of Clinical Medicine and Surgery, Respiratory Medicine Unit, Federico II University of Naples, Azienda Ospedali dei Colli, P.O. Monaldi, Naples, Italy.; 2. Department of Diagnostic Imaging, Section of General Radiology, Azienda Ospedali dei Colli, P.O. Monaldi, Naples, Italy.

## TO THE EDITOR:

**Figure 1:**
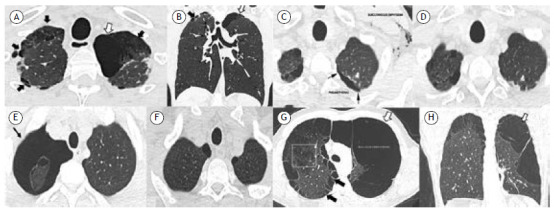
Axial and coronal unenhanced thin-slice chest HRCT scans (A and B, respectively) in a 25-year-old male cannabis smoker, showing a large (5 cm) bulla (white arrows), sharply demarcated by a thin wall in the left lung apex, together with multiple small blebs adjacent to the pleura and small areas of paraseptal and centrilobular emphysema in both lung apices (black arrows). Axial unenhanced thin-slice chest HRCT scans (C and D, respectively) in a 45-year-old male cannabis smoker, showing left pneumothorax (arrows in C) with marked bilateral apical subcutaneous emphysema and sparse areas of paraseptal emphysema along with small blebs and rare bullae that resolved after drainage. Axial unenhanced thin-slice chest HRCT scans (E and F, respectively) in a 23-year-old male cannabis smoker, showing extensive pneumothorax on the right side (black arrow in E) with some blebs adjacent to the visceral anterior pleura and small areas of paraseptal emphysema on the same side, with complete resorption of pneumothorax after chest drainage. Axial unenhanced thin-slice chest HRCT scan (G) in a 32-year-old male cannabis smoker, showing extensive alterations due to destruction of lung tissue on both sides with a coarse bullous formation on the left (white arrow). Coronal multiplanar reconstruction (H) of the same patient showing the bullous formation occupying the upper and middle thirds of the left hemithorax, with subtle septation. In the right lung (visible in G), there were marked changes from paraseptal and centrilobular emphysematous damage (black arrows and square outline, respectively).

Cannabis, also known as marijuana, is the most abused illicit psychoactive substance worldwide. Because it has been legalized in some countries, its use is expected to increase. Cannabis can be consumed by smoking, by vaping, by infusion, by ingestion, or as a tincture. When inhaled, it can induce lung injury. We evaluated six young male cannabis smokers who presented with lung involvement between September 2022 and December 2023. All of the patients gave written informed consent. The mean age was 31.6 years, and the mean BMI was 21.3 kg/m^2^. Collectively, the patients had been cannabis users for 5-20 years, smoking 1-10 cannabis cigarettes (“joints”) per day. None was a tobacco smoker or had any chronic lung disease. All tested negative for HIV and alpha-1 anti-trypsin deficiency. General blood examinations were unremarkable. All patients underwent unenhanced thin-slice HRCT of the chest. One of the patients, a 25-year-old, reported a six-month history of exertional dyspnea. He had smoked 4-5 joints/day for five years. Representative chest CT images of that patient are shown in Figures 1A and 1B. Four of the other patients (age range, 23-45 years) had smoked 2-5 joints per day for 5-20 years and presented to the emergency room with acute chest pain and dyspnea due to spontaneous pneumothorax requiring drainage (Figure 1, C-F). The remaining patient was a 32-year-old heavy smoker of cannabis with long-standing exertional dyspnea (Figures 1G and 1H). The chest CT findings are summarized in Table 1. In two patients without pneumothorax, a mild restrictive ventilatory pattern was detected, together with a mild reduction in single-breath DL_CO_. None of the patients had respiratory failure. Because lung damage from cannabis use is still poorly recognized, it is underdiagnosed. The clinical presentation can be insidious, with pneumothorax occurring as an apparently idiopathic spontaneous entity. The definition of cannabis lung is imaging-based, including the presence of large bullae with predominant apical involvement in individuals with a history suggestive of cannabis smoking.^1^ However, these features are nonspecific because they have been most widely associated with concomitant tobacco use in anecdotal case reports. Therefore, there is still a lack of studies characterizing radiological findings attributed exclusively to cannabis use. In the first attempt to do so in a systematic manner, Murtha et al. recently described the chest CT findings of 56 cannabis smokers.^2^ However, even in that setting, 50 of the individuals analyzed were also tobacco smokers. The authors found that paraseptal emphysema and blebs were the most distinctive features. Those alterations, together with the involvement of chemical and physical factors related to cannabis use, are thought to lead to bullae formation. The mechanism of bullae formation seems to involve cannabis-induced lung toxicity in combination with pleural pressure swings and airway barotrauma, of which the last two are caused by the high inspiratory pressures resulting from cannabis smoking. In comparison with tobacco smokers, cannabis smokers take larger puffs, inhale more deeply, and hold their inhalations longer.^3^ Unlike cigarettes, joints are typically unfiltered, which increases tar deposition and carboxyhemoglobin formation by 4-5 times.^3^ Cannabis-induced lung injury can go beyond the mere formation of bullae. Short-term exposure to cannabis can lead to bronchodilation, due to the effect of delta-9-tetrahydrocannabinol (the main psychoactive component of cannabis). Long-term exposure is associated with respiratory symptoms (cough, phlegm production, and wheezing) but not necessarily with bronchial obstruction.^4^ ^,^ ^5^ There is evidence that airway inflammation, increased secretions, and bronchial remodeling occur in cannabis smokers.^6^ ^,^ ^7^ Murtha et al.^2^ also stated that chest HRCT evidence of bronchial thickening, mucoid impaction, and bronchiectasis can be expected to be found in cannabis smokers. Inspired by these observations and in an attempt to provide data that enable precise interpretation, we exclusively evaluated cannabis smokers who were not tobacco users. With the exception of gynecomastia, all of the radiological findings described by Murtha et al.^2^ were represented in our patients, suggesting that they had an exclusively cannabis-related origin. Another element that characterized our study sample was the low mean age (31.6 years), which further underscores the need to focus on this entity whose evolution can be insidious and potentially disabling over time. Spontaneous pneumothorax was the mode of disease presentation in four of the cases presented here. Although this complication is already known in cannabis smokers, the causal association often goes uninvestigated because the evolution of spontaneous pneumothorax can be subtle. This insidious aspect is partly attributable to the young age and the long-line phenotype of subjects with idiopathic spontaneous pneumothorax, which can be confused with that of undeclared cannabis users with lung involvement, suggesting that such a clinical scenario is more common than expected. In our case series, the combination of young age with the extent and type of radiological damage was immediately suspicious for a potential association with cannabis use, which was initially denied by all of the patients. Related clinical aspects should also not be neglected in such patients, especially because they can be used in order to exclude other forms of tobacco-induced disorders with which cannabis lung is confused. In addition, some considerations on the potential for disease evolution merit greater attention. Although the current data are inconclusive, the risk of developing lung cancer in the long term should not be ignored.^6^ ^,^ ^8^ There is no doubt that our report is limited by the small size of our sample. A further limitation is the lack of complete lung function data, mainly due to the caution imposed by the extensive lung damage or to the need for pleural drainage positioning. However, our effort might help shed light on an intriguing topic that merits further exploration in larger series through the same systematic approach. In that sense, our contribution aims to sensitize the scientific and medical community to be more suspicious when faced with an entity whose clinical-radiological presentation and course can be even more insidious if the habits of the patient are not properly investigated.

**Table 1:** Synopsis of cannabis-related chest HRCT findings.

Finding	Case 1	Case 2	Case 3	Case 4	Case 5	Case 6
Emphysema	x	x	x	x	x	x
Paraseptal emphysema	x	x	x	x	x	x
Blebs/bullae	x	x	x	x	x	x
Bronchial thickening	x	x	x	-	x	x
Bronchiectasis	-	x	-	-	-	-
Mucoid impaction	x	x	x	x	x	-
Gynecomastia	-	-	-	-	-	-
Coronary artery calcification	-	-	-	-	-	x
